# Recurrent Tuberculosis of Greater Trochanter and Its Bursa

**DOI:** 10.1155/2013/570956

**Published:** 2013-08-12

**Authors:** Keshav S. Shenoy, Santosh S. Jeevannavar, Prasanna Baindoor, Sunil Mannual, Savith V. Shetty

**Affiliations:** Department of Orthopaedics, Shri Dharmasthala Manjunatheshwara College of Medical Sciences & Hospital, Manjushree Nagar, Sattur, Dharwad, Karnataka 580009, India

## Abstract

A 65-year-old female had a history of tuberculosis of the left greater trochanter 30 years ago. She underwent 6 months of chemotherapy after which the disease healed completely. Currently she presented to us with pain and swelling on the lateral aspect of left hip of 2-month duration. Clinical and radiological findings were suggestive of a recurrence. Biopsy was conclusive for tuberculosis. She was successfully treated with debridement and curettage with chemotherapy for 1 year. Recurrent tuberculosis of the greater trochanter is rare and should be aggressively treated.

## 1. Introduction

Primary tubercular involvement of the greater trochanter and its overlying bursa is rare and accounts for 1-2% of all musculoskeletal tuberculosis [1]. The disease spreads to the trochanter most commonly by hematogenous means. The patient presents with lateral hip pain of prolonged duration with or without limp. Long-standing disease may involve the hip joint [2]. The differential diagnoses include tumors, septic bursitis, idiopathic bursitis, and osteochondritis [3]. Newer diagnostic modalities like CT and MRI scans help in early diagnosis and delineating the lesion better. 

 Tubercular lesions are known to recur in people with poor immune function, especially elderly. We describe a 65-year-old lady with primary tuberculosis of the left greater trochanter and its overlying bursa treated successfully by chemotherapy, who presented to us with recurrent disease after 30 years. Excision of the bursa, curettage of the lytic lesion and antitubercular chemotherapy was curative. At 2-year followup, patient were asymptomatic and the disease had completely healed. 

## 2. Case Report

 65-year-old lady presented to us with a history of pain and swelling on the lateral aspect of left hip of 2-month duration. Both pain and swelling were gradual in onset and progressive in nature. Pain was constant, dull aching type, increasing on weight bearing and partially relieved with analgesics. There was no history of trauma, fever, or other constitutional symptoms. She did not have any other comorbid illness. 

 Thirty years back patient had pain and swelling on the lateral aspect of left thigh along with a discharging sinus. She was diagnosed to have tuberculosis of the left trochanteric bursa based on the clinical and radiological findings. Short course antitubercular chemotherapy for 6 months was curative and the patient was asymptomatic since then.

### 2.1. Clinical Examination

**Figure 1:**
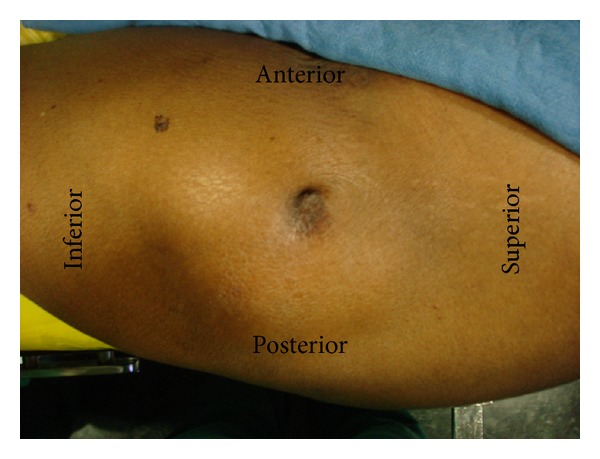
Clinical photograph of the lateral aspect of the left hip region showing a well-defined swelling overlying the left greater trochanter with an adherent dimple scar over it.

General physical examination was unremarkable. On local examination, there was a fluctuant swelling measuring about 8 × 4 cm on the lateral aspect of the left hip, overlying the greater trochanter, with a dimple of healed sinus adherent to it (Figure 1). There was minimal local warmth and tenderness. Range of motion of left hip was normal and the patient walked with an antalgic gait without any support.

### 2.2. Investigations

**Figure 2:**
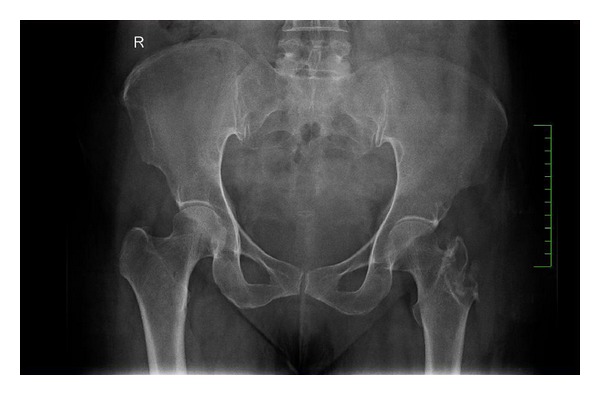
X-ray of the pelvis with both hips showing a well-defined eccentric osteolytic lesion involving the left greater trochanter with an enlarged soft tissue shadow adjacent to it.

**Figure 3:**
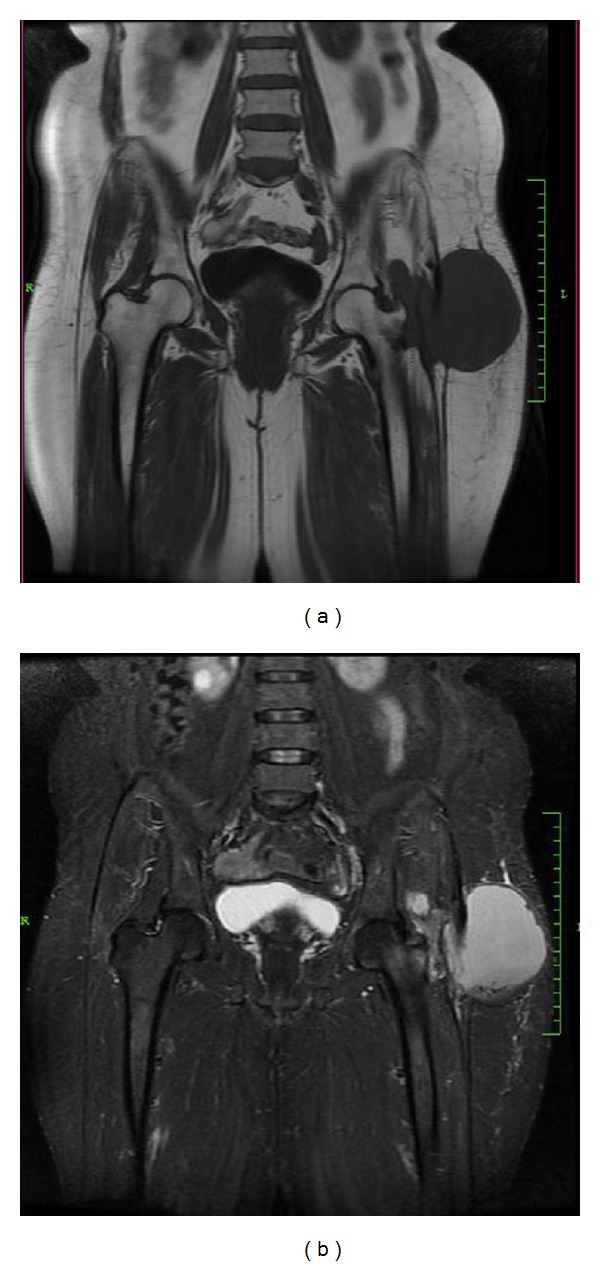
Coronal T1- and T2-weighted MRI image of the pelvis showing focal areas of altered marrow signal intensity changes appearing hypointense on T1-weighted image and hyperintense on T2-weighted image, suggestive of infective lesion. A large localized fluid collection is seen in the subcutaneous tissues of the lateral aspect of the left thigh.

**Figure 4:**
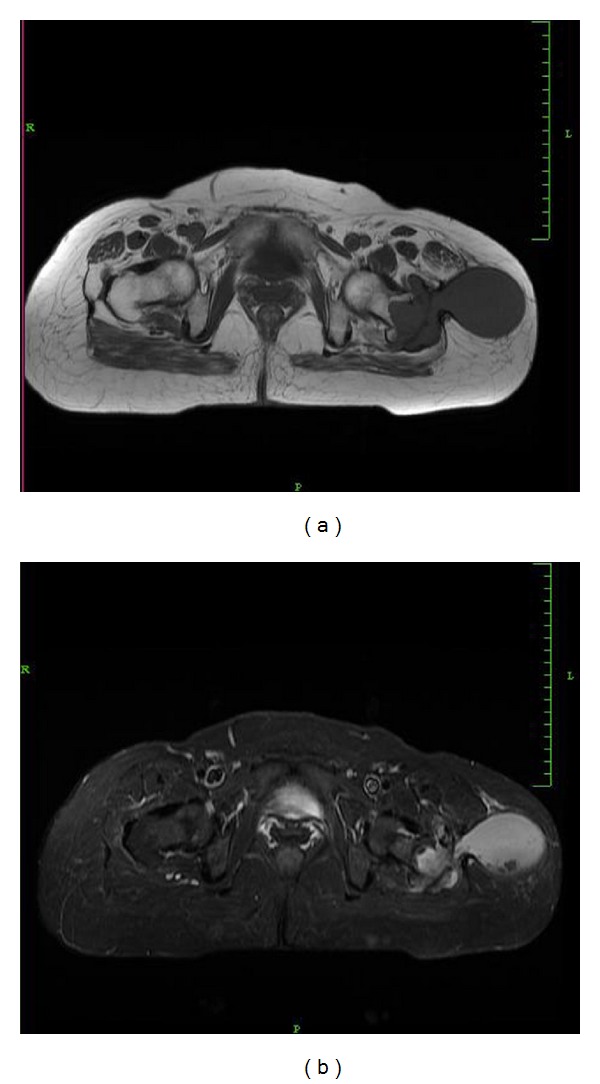
Axial T1- and T2-weighted MRI image of the pelvis showing the abscess extending from the left greater trochanter into the subcutaneous tissues laterally through a small defect in the deep fascia resembling a dumb-bell-shaped abscess.

Her laboratory investigations showed normal hemoglobin (12.3 g/dL) with normal total counts (9,960 cells/mm^3^) and differential counts (neutrophils 63%, lymphocytes 27%, monocytes 4%, eosinophils 6%, and basophils 0%). Erythrocyte sedimentation rate was high (50 mm in 1st hour). Liver functions, renal functions, and blood sugar levels were normal. Chest X-ray was normal. X-ray of the pelvis with both hips showed a lytic lesion of the left greater trochanter with sharp margins suggestive of benign tumour or infection (Figure 2). MRI scan of pelvis revealed a focal osteolytic lesion with cortical destruction in the left greater trochanter and an enlarged trochanteric bursa with an enhancing wall suggestive of an abscess. The abscess extended into the subcutaneous tissues laterally through a small defect in the deep fascia, resembling a dumb-bell-shaped abscess and contained multiple small loose bodies (Figures 3 and 4). FNAC of the lesion was inconclusive.

### 2.3. Treatment

**Figure 5:**
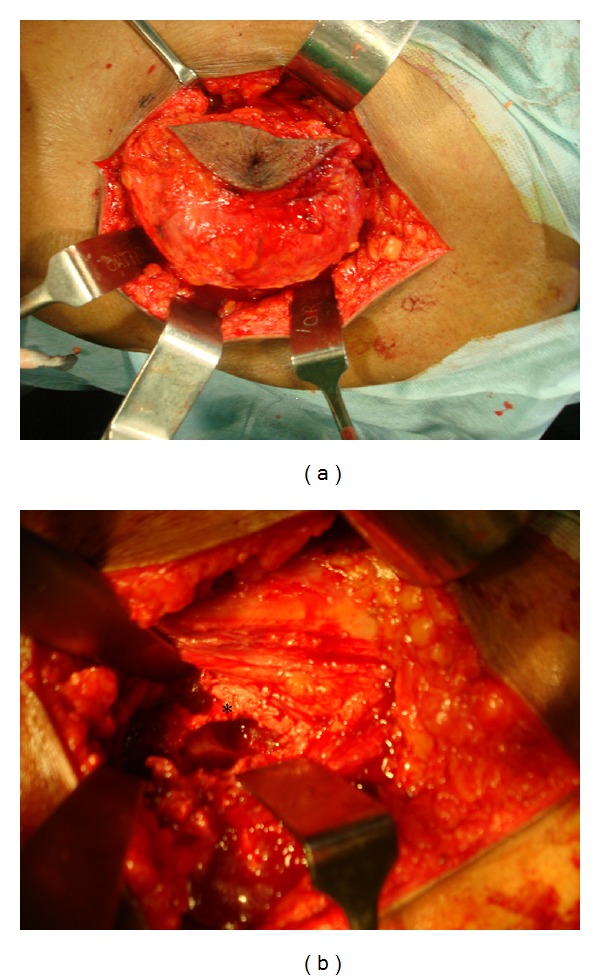
(a) Intraoperative photograph after the dissection of the swelling along with the dimple scar before it was excised in toto. (b) Osteolytic cavity of the greater trochanter (*) after it was thoroughly debrided.

**Figure 6:**
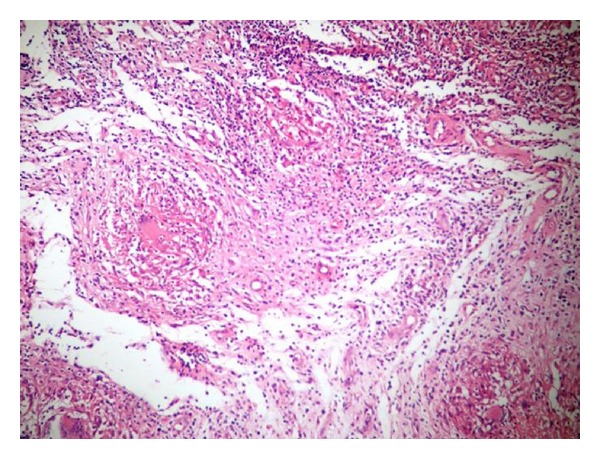
Hematoxylin and Eosin stained biopsy specimen showing typical areas of caseating necrosis surrounded by epithelioid cells and Langerhan's giant cells.

Excision biopsy and debridement of the osteolytic lesion of the greater trochanter were done by lateral approach to the proximal femur. The entire abscess was excised in toto and the osteolytic cavity of the greater trochanter was thoroughly debrided. Thick yellow pus was noted in the abscess cavity. The attachment of the glutei was found to be intact and not violated during the surgery (Figures 5(a) and 5(b)). The abscess wall was sent for biopsy and the pus was sent for routine culture, ZN staining, Gram staining, and culture for tuberculosis. Postoperative period was uneventful. Routine culture revealed no growth. Gram staining and ZN staining did not show any organisms and culture for tuberculosis was negative at the end of 6 weeks. Biopsy of the specimen showed many caseating granulomas consisting of epithelioid cells and Langerhan's giant cells suggestive of tuberculosis (Figure 6).

Patient was started on Category I antitubercular chemotherapy (2H_3_R_3_Z_3_E_3_ + 4H_3_R_3_) for 12 months. She was followed up regularly and, at each followup, progressive improvement was noted clinically, hematologically and radiologically. At 3-year followup, the disease had completely healed; the patient was totally asymptomatic and walking with a normal, pain-free gait. Repeat MRI of the pelvis was done at 2 years and it showed subsidence of the marrow edema in the trochanter and resolution of swelling, consistent with healing.

## 3. Discussion

Hip is the second most frequent site of osteoarticular tuberculosis after the spine [2]. The disease may start in the femoral head, neck, joint cavity, and acetabulum or in the greater trochanter and its overlying bursa. Of these, the primary involvement of greater trochanter and its bursa are the rarest and account for 1-2% of all cases of osteoarticular tuberculosis. Trochanteric bursa, though, is the most common site of tuberculous bursitis [4]. There is no predilection for any particular age group or sex. The disease is usually multifocal though rarely it may affect only the greater trochanter and its bursa. In our case, there was primary involvement of the greater trochanter and its bursa, as there was no present or past evidence of pulmonary involvement.

The patient presents with pain with or without swelling on the lateral aspect of the hip overlying the greater trochanter. Chronic, untreated cases may present with discharging sinus on the lateral aspect of the hip; however, in our case, pain and increasing swelling over the greater trochanter were the presenting complaints. There was evidence of healed dimple scar over the swelling. Involvement of hip joint is very rare. Fever and other constitutional symptoms may be present in up to 30% of the cases [2]. Radiographs may be normal initially but may show erosion or lytic lesion of the greater trochanter in later stages. MRI may be the investigation of choice as it can clearly demonstrate, in multiple planes, the extent of soft tissue mass along with the bony involvement [5]. However, it is not specific for tuberculosis [4]. We feel that MRI, in addition to defining the extent of the lesion, will also act as a guide in surgical planning for recurrent cases. In our case the origin of the swelling was defined accurately as the extent of bony involvement of the trochanter region which is quiet important in surgical planning for recurrent cases. The differential diagnoses include tumours, septic bursitis, chronic pyogenic osteomyelitis, and idiopathic trochanteric bursitis [3]. The diagnosis can be established either by culture or biopsy, which shows caseating granulomas lined by epithelioid cells and Langerhan's giant cells.

A review of literature shows a few case reports and case series of primary tuberculosis of the greater trochanter [1, 3, 5–11]. In most of these cases, the diagnosis was established by biopsy and complete excision of the lesion along with chemotherapy was curative. Most of these reports are from the previous decade and they undermine the need for investigations like MRI in diagnosing the condition and delineating the extent of lesion.

Tuberculosis is known to recur in patients with poor immune status like in the elderly. Sastre et al. have reported a case of reactivation of trochanteric tuberculosis after previous surgical drainage done in the prechemotherapy era. They achieved a good result with wide excision and curettage of the bone along with chemotherapy for 9 months [3]. Yamamoto et al. have reported a case of tubercular bursitis of the greater trochanter occurring 51 years after tubercular nephritis. They too achieved a good result with total excision and chemotherapy [12]. In our case, the patient had pain swelling and discharging sinus on the lateral aspect of the thigh 30 years back. She was diagnosed to have tuberculosis of the greater trochanteric bursa based on clinical and radiographic findings. Chemotherapy for 6 months was curative and the discharging sinus healed as a dimple scar. Following this, she was totally asymptomatic till she presented to us when she complained of pain and swelling on the lateral side of the thigh of 2-month duration. Keeping in mind the previous history of tuberculosis, a provisional diagnosis of recurrence of tuberculosis of the greater trochanter and its bursa was made. After the radiological investigations, she underwent total excision of the infected bursa and curettage of the lytic lesion in the greater trochanter as outlined in the MRI. Biopsy of the lesion confirmed the diagnosis of tuberculosis. Patient was treated with extended chemotherapy for 12 months following which the disease healed and she was asymptomatic at 3-year followup.

## 4. Conclusion 

Our case demonstrates that an old healed tubercular lesion can recur, especially in the elderly, in whom the immune status is waning. MRI is useful in delineating the extent of the lesion especially in sites of rare affection like the greater trochanter and also guides in surgical planning. MRI may also be useful to monitor the response to the antitubercular chemotherapy in such cases. Total excision of the lesion along with antitubercular chemotherapy may be associated with lesser recurrence rates as compared to chemotherapy alone.
